# Supplementary material for: Association between Coffee Consumption, Caffeine Intake, and Metabolic Syndrome Severity in Patients with Self-Reported Rheumatoid Arthritis: National Health and Nutrition Examination Survey 2003–2018
Source: Nutrients. 2022 Dec 26;15(1):107. doi: 10.3390/nu15010107 (PMC9824592; doi:10.3390/nu15010107)
Supplement: Supplementary file 1 [file nutrients-15-00107-s001.zip › nutrients-2022169-supplementary.pdf]

Supplementary Materials

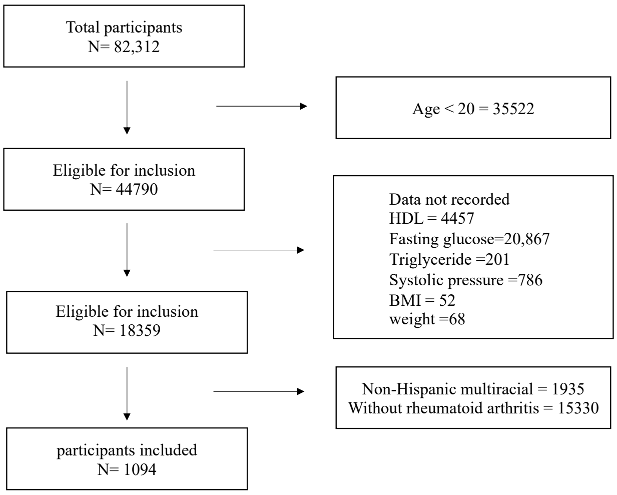

Figure S1. Cohort Construction Flowchart. HDL, high-density lipoprotein; BMI, body mass index.

Table S1. Characteristics of the study population grouped by decaffeinated coffee intake.

Characters	Nondrinker (n=895)	0-2 cups/day (n=124)	>2cups/day (n=75)	p value
Age	59.63±0.55	66.54±1.36	67.45±1.07	<0.001
BMI (kg/m^2)	30.33±0.29	28.88±0.72	30.36±0.87	0.14
PIR	2.88±0.06	3.07±0.2	3.16±0.19	0.15
Energy (kcal)	1981.69±33.73	1823.36±59.43	1793.56±89.17	0.018
MetS Z-score	0.42±0.04	0.35±0.11	0.38±0.15	0.74
SBP (mmHg)	126.51±0.77	129.89±2.29	132.02±3.4	0.023
Insulin (μU/mL)	13.58±0.59	12.5±1.38	13.69±2.51	0.75
Total cholesterol (mg/dl)	5.13±0.05	5.01±0.11	5.47±0.14	0.15
HDL (mg/dl)	54.59±0.67	56.23±1.49	60.04±2.59	0.011
Fasting glucose (mg/dl)	109.21±1.15	108.73±3.13	107.52±2.98	0.89
Triglyceride (mg/dl)	143.56±4.47	130.67±7.3	131.97±8.89	0.28
HOMA-IR	72.64±5.22	66.12±9.52	70.02±14.65	0.86
Sex				0.12
Male	39.89(0.37-0.43)	37.1(0.29-0.46)	34.67(0.25-0.46)	
Female	60.11(0.57-0.63)	62.9(0.54-0.71)	65.33(0.54-0.75)	
Race				0.096
Mexican American	9.94(0.08-0.12)	14.52(0.09-0.22)	5.33(0.02-0.13)	
Other Hispanic	7.04(0.06-0.09)	9.68(0.06-0.16)	4(0.01-0.12)	
Non-Hispanic White	60.89(0.58-0.64)	51.61(0.43-0.6)	82.67(0.72-0.9)	
Non-Hispanic Black	22.12(0.2-0.25)	24.19(0.17-0.33)	8(0.04-0.17)	
Education				0.21
Less Than 9th Grade	9.83(0.08-0.12)	16.94(0.11-0.25)	16(0.09-0.26)	
9-11th Grade	16.54(0.14-0.19)	8.87(0.05-0.15)	10.67(0.05-0.2)	
High School	24.92(0.22-0.28)	24.19(0.17-0.33)	21.33(0.13-0.32)	
Some College	28.6(0.26-0.32)	28.23(0.21-0.37)	26.67(0.18-0.38)	
College Graduate	20.11(0.18-0.23)	21.77(0.15-0.3)	25.33(0.17-0.36)	

Alcoholic >4 drinks				<0.001
Yes	78(0.75-0.82)	84(0.72-0.92)	66(0.54-0.77)	
No	7(0.05-0.09)	2(0.00-0.05)	2(0.00-0.08)	
Not recorded	15(0.12-0.18)	14(0.07-0.26)	32(0.21-0.45)	
Glucocorticoid				0.03
YES	0.4(0.00-0.02)	0	2(0.00-0.13)	
No	97.8(0.96-0.99)	100	93(0.83-0.97)	
Not recorded	1.8(0.01-0.03)	0	5(0.02-0.14)	
Smoke				0.49
Yes	52.29(0.49-0.56)	45.97(0.37-0.55)	66.67(0.55-0.76)	
No	47.49(0.44-0.51)	54.03(0.45-0.63)	33.33(0.24-0.45)	
Not recorded	0.22(0-0.01)	0	0	

Data are presented as means (SE) for continuous measures, and as percentage (95% confidence interval) for categoric measures.; p value was calculated by weighted linear regression model for continuous variables and chi-square test for categorical variables, respectively. BMI, body mass index; PIR, poverty-income ratio; SBP, systolic blood pressure; HDL, high density lipoprotein; SE (standard error); CI, confidence interval.

Table S2. Characteristics of the study population grouped by total caffeine intake.

Characters	<100mg/day (n=509)	100-200mg/day (n=310)	>200mg/day (n=275)	p value
Age	63.24±0.73	61.85±0.88	56.96±0.92	<0.001
BMI (kg/m²)	30.07±0.41	30.33±0.44	30.24±0.52	0.88
PIR	2.73±0.09	3.04±0.11	3.04±0.11	0.0036
Energy (kcal)	1826.7±42.34	1957.83±50.67	2107.67±59.32	<0.001
MetS Z-score	0.42±0.05	0.49±0.07	0.34±0.07	0.18
SBP (mmHg)	130.37±1.16	125.3±1.24	125.03±1.33	<0.001
Insulin (μU/mL)	12.78±0.65	14.53±1.3	13.43±0.93	0.24
Total cholesterol (mg/dl)	5.14±0.06	5.17±0.1	5.13±0.07	0.89
HDL (mg/dl)	56.66±0.96	53.25±1.07	54.98±1.14	0.016
Fasting glucose (mg/dl)	108.75±1.5	110.37±2.12	108.19±1.78	0.65
Triglyceride (mg/dl)	133.92±4.16	152.19±9.94	141.3±6.15	0.037
HOMA-IR	66.96±4.28	83.56±13.56	67.3±5.21	0.11
Sex				<0.001
Male	52.29(0.49-0.56)	45.97(0.37-0.55)	66.67(0.55-0.76)	
Female	47.49(0.44-0.51)	54.03(0.45-0.63)	33.33(0.24-0.45)	
Race				<0.001
Mexican American	11.39(0.09-0.14)	10.97(0.08-0.15)	6.91(0.04-0.11)	
Other Hispanic	6.68(0.05-0.09)	9.03(0.06-0.13)	5.82(0.04-0.09)	
Non-Hispanic White	50.49(0.46-0.55)	62.9(0.57-0.68)	79.64(0.74-0.84)	
Non-Hispanic Black	31.43(0.28-0.36)	17.1(0.13-0.22)	7.64(0.05-0.11)	
Education				0.001
Less Than 9th Grade	14.54(0.12-0.18)	10(0.07-0.14)	5.82(0.04-0.09)	
9-11th Grade	14.54(0.12-0.18)	17.42(0.14-0.22)	14.18(0.11-0.19)	
High School	25.93(0.22-0.3)	23.87(0.19-0.29)	22.91(0.18-0.28)	
Some College	26.13(0.22-0.3)	27.42(0.23-0.33)	33.82(0.28-0.4)	
College	18.86(0.16-0.22)	21.29(0.17-0.26)	23.27(0.19-0.29)	
Alcoholic >4 drinks				<0.001
No	3(0.02-0.05)	5(0.03-0.09)	11(0.07-0.17)	
Yes	81(0.77-0.85)	79(0.73-0.84)	73(0.66-0.79)	

Not recorded	16(0.12-0.20)	16(0.12-0.22)	16(0.12-0.21)	
Glucocorticoid				0.13
No	98.6(0.97-0.99)	97(0.94-0.98)	97(0.93-0.99)	
YES	0.4(0.00-0.03)	0	1(0.00-0.05)	
Not recorded	1(0.00-0.02)	3(0.02-0.06)	2(0.01-0.05)	
Smoke				<0.001
Yes	42.04(0.38-0.46)	52.58(0.47-0.58)	72(0.66-0.77)	
No	57.76(0.53-0.62)	47.42(0.42-0.53)	27.64(0.23-0.33)	
Not recorded	0.2(0-0.01)	0	0.36(0-0.03)	

Data are presented as means (SE) for continuous measures, and as percentage (95% confidence interval) for categoric measures.; p value was calculated by weighted linear regression model for continuous variables and chi-square test for categorical variables, respectively. BMI, body mass index; PIR, poverty-income ratio; SBP, systolic blood pressure; HDL, high density lipoprotein; SE (standard error); CI, confidence interval.

Table S3. Weighted linear regression for MetS z-score in coffee, decaffeinated coffee and caffeine intake group after fully adjustment.

characteristic	Coffee, model 3		Decaffeinated coffee, model 3		Caffeine intake, model 3	
	**Coefficient (95% CI)**	***P***	**Coefficient (95% CI)**	***P***	**Coefficient (95% CI)**	***P***
Age	0.00(0.00-0.00)	0.96	0.00(0.00-0.01)	0.76	0.00(0.00-0.00)	0.94
Education (ref.= Less Than 9th Grade)						
9-11th Grade	-0.17(-0.47-0.12)	0.25	-0.18(-0.47-0.12)	0.24	-0.17(-0.46-0.13)	0.27
High School	-0.19(-0.46-0.08)	0.18	-0.20(-0.47-0.07)	0.15	-0.18(-0.45-0.09)	0.18
Some College	-0.27(-0.55-0.00)	0.05	-0.28(-0.56 to-0.01)	0.05	-0.27(-0.54-0.01)	0.06
College	-0.26(-0.54-0.03)	0.08	-0.26(-0.55-0.02)	0.07	-0.25(-0.54-0.03)	0.18
PIR	-0.08(-0.12 to -0.03)	<0.001	-0.08(-0.12 to -0.03)	<0.001	-0.08(-0.12 to -0.03)	<0.001
Energy (kcal)	0.00(0.00-0.00)	0.009	0.00(0.00-0.00)	0.01	0.00(0.00-0.00)	0.009
LDL (mg/dl)	0.04(-0.02-0.11)	0.16	0.05(-0.02-0.11)	0.15	0.04(-0.02-0.10)	0.19
Insulin (μU/mL)	0.01(0.01-0.02)	0.001	0.01(0.01-0.02)	0.001	0.01(0.01-0.02)	0.001
Waist Circumference (cm)	0.03(0.03-0.04)	<0.001	0.03(0.03-0.04)	<0.001	0.03(0.03-0.04)	<0.001
Smoke (ref. = Yes)						
No	-0.07(-0.17-0.04)	0.22	-0.05(-0.15-0.06)	0.4	-0.05(-0.16-0.05)	0.32
Not recorded	-0.01(-0.59-0.57)	0.97	-0.01(-0.60-0.58)	0.98	0.01(-0.59-0.61)	0.98
Alcoholic >4 drinks (ref. = No)						
Yes	-0.17(-0.42-0.08)	0.19	-0.18(-0.43-0.08)	0.17	-0.17(-0.42-0.08)	0.19
Not recorded	0.03(-0.12-0.19)	0.66	0.05(-0.10-0.20)	0.49	0.04(-0.11-0.19)	0.59
Glucocorticoid (ref. = No)						
YES	0.01(-0.62-0.64)	0.98	0.01(-0.59-0.62)	0.97	0.00(-0.61-0.61)	0.99
Not recorded	-0.23(-0.44 to -0.03)	0.03	-0.25(-0.47 to -0.04)	0.02	-0.27(-0.48 to-0.06)	0.01
Coffee (ref. =nondrinkers)						
0-2 cups	-0.07(-0.22-0.07)	0.33	-	-	-	-
>2 cups	-0.13(-0.24 to -0.01)	0.04	-	-	-	-

Decaffeinated Coffee						
(ref. =nondrinkers)						
0-2 cups	-	-	-0.01(-0.19-0.16)	0.89	-	-
>2 cups	-	-	-0.15(-0.36-0.05)	0.15	-	-
Caffeine intake						
(ref. = 0-100 mg)						
100-200mg	-	-	-	-	0.02(-0.12-0.15)	0.79
>200mg	-	-	-	-	-0.06(-0.19-0.06)	0.31

Adjusted model included age, education level, PIR, total energy intake, LDL, fasting insulin, waist circumference, smoke and alcohol exposure, use of glucocorticoid. PIR, poverty-income ratio; LDL, low density lipoprotein; CI, confidence interval; Ref., reference; A β-coefficient was calculated by weighted linear regression for MetS z-score.

Table S4. Weighted logistic regression for prevalence of MetS in coffee intake group.

Coffee intake	Model 1		Model 2		Model 3	
	OR (95% CI)	*P*	OR (95% CI)	*P*	OR (95% CI)	*P*
Coffee (ref. =nondrinkers)						
0-2 cups	1.15(0.66-2.01)	0.48	0.99(0.56-1.75)	0.99	1.07(0.59-1.94)	0.82
>2 cups	1.14(0.71-1.83)	0.53	0.90(0.54-1.50)	0.69	1.03(0.59-1.82)	0.91

Model 1 did not adjust for any other variables. Model 2 was adjusted for age, sex, education level and PIR. Model 3 was adjusted for age, sex, education level, PIR, total energy intake, LDL, fasting insulin, waist circumference, smoke and alcohol exposure, use of glucocorticoid; OR, odds ratio; LDL, low density lipoprotein; CI, confidence interval; Ref., reference; An Odd ratio was calculated by weighted logistic regression for the prevalence of MetS.

Table S5. Relationship between the components of the z-score and coffee intake in high- and low- risk group.

Coffee(ref.=nondrinker)	Low-risk group		High-risk group	
characteristic	Coefficient (95% CI)	*P*	Coefficient (95% CI)	*P*
0-2 cups/day				
BMI (kg/㎡)*	-0.36(-1.20-0.48)	0.4	-0.63(-1.49-0.22)	0.15
HDL (mg/dl) *	-2.7(-7.99-2.58)	0.32	-1.0(-3.6-1.6)	0.45
Fasting glucose (mg/dl) **	-0.05(-0.20-0.11)	0.56	-0.07(-0.45-0.31)	0.72
Triglyceride (mg/dl) *	-3.49(-14.15-7.17)	0.52	5.65(16.16-27.45)	0.61
SBP (mmHg)	-3.45(-10.37-3.47)	0.33	-0.17(-4.26-3.32)	0.69
>2 cups/day				
BMI (kg/㎡)*	-0.13(-0.77-0.52)	0.7	-0.87(-1.73to -0.01)	0.048
HDL (mg/dl) *	-3.70(-8.55-1.16)	0.14	0.69(-1.90-3.29)	0.6
Fasting glucose (mg/dl) **	0.14(0.01-0.28)	0.03	-0.22(-0.60-0.16)	0.26
Triglyceride (mg/dl) *	4.19(-6.35-14.73)	0.44	13.31(-16.16-27.46)	0.29
SBP (mmHg)	-9.96(-14.17 to -5.74)	<0.01	-0.17(-4.26-3.93)	0.94

* Adjusted for age, gender, education level, total energy intake, PIR, HOMA-IR, smoke status, triglyceride, total cholesterol, waist circumference and use of glucocorticoid. **Adjusted for age, gender, education level, total energy intake, PIR, smoke status, triglyceride, total cholesterol, waist circumference and use of glucocorticoid. ***Adjusted for age, gender, education level, PIR, triglyceride, total cholesterol, waist circumference and use of glucocorticoid. BMI, body mass index; HDL, high density lipoprotein; CI, confidence interval; Ref., reference; A β-coefficient was calculated by weighted linear regression for BMI, HDL, fasting glucose, triglyceride and SBP, respectively.

Table S6. Sensitivity analysis for MetS z-score in all participants, low-risk group and high-risk group.

characteristic	Total	Low-risk group	High-risk group

	Coefficient (95% CI)	*P*	Coefficient (95% CI)	*P*	Coefficient (95% CI)	*P*
Coffee (ref. =nondrinkers)						
0-2 cups	-0.13(-0.30-0.04)	0.1	-0.13(-0.25 to -0.02)	0.02	-0.09(-0.29-0.11)	0.37
>2 cups	-0.20(-0.39 to -0.01)	0.04	0.08(-0.06-0.21)	0.27	-0.24(-0.49-0.00)	0.048
Decaffeinated Coffee (ref. =nondrinkers)						
0-2 cups	-0.05(-0.23-0.13)	0.6	-0.10(-0.29-0.09)	0.31	-0.14(-0.33-0.06)	0.17
>2 cups	-0.21(-0.42-0.00)	0.05	-0.05(-0.23-0.13)	0.62	-0.10(-0.35-0.14)	0.41
Caffeine intake (ref. = 0-100 mg)						
100-200mg	0.10(-0.07-0.26)	0.26	0.03(-0.09-0.15)	0.62	0.10(-0.10-0.29)	0.33
>200mg	0.05(-0.14-0.24)	0.6	-0.18(-0.33 to -0.03)	0.02	0.07(-0.16-0.31)	0.54

Adjusted model included age, education level, PIR, total energy intake, LDL, fasting insulin, waist circumference, smoke and alcohol exposure, use of glucocorticoid, decaffeinated coffee and daily caffeine intake. BMI, body mass index; HDL, high density lipoprotein; CI, confidence interval; Ref., reference; A β-coefficient was calculated by weighted linear regression for MetS z-score.
